# Risk of autoimmune diseases in patients with RASopathies: systematic study of humoral and cellular immunity

**DOI:** 10.1186/s13023-021-02050-6

**Published:** 2021-10-02

**Authors:** M. A. Siano, V. Marchetti, S. Pagano, F. Di Candia, M. Alessio, D. De Brasi, A. De Luca, V. Pinna, S. Sestito, D. Concolino, M. Tartaglia, P. Strisciuglio, V. D’Esposito, S. Cabaro, G. Perruolo, P. Formisano, D. Melis

**Affiliations:** 1 Department of Medicine, Surgery and Dentistry, “Scuola Medica Salernitana”, Salerno, Italy; 2 Dipartimento di Scienze Mediche Traslazionali- Sez. di Pediatria, Università degli Studi di Napoli “Federico II”, Napoli, Italy; 3 Dipartimento di Pediatria, A.O.R.N. “Santobono-Pausillipon”, Napoli, Italy; 4 Molecular Genetics Unit, Fondazione Casa Sollievo della Sofferenza, IRCCS, San Giovanni Rotondo, Foggia Italy; 5 Dipartimento di Medicina Clinica e Sperimentale, Università “Magna Graecia” di Catanzaro, Catanzaro, Italy; 6 Genetics and Rare Diseases Research Division, Ospedale Pediatrico Bambino Gesù, Rome, Italy; 7 Dipartimento di Scienze Mediche Traslazionali, Università degli Studi di Napoli “Federico II” & Istituto di Endocrinologia e Oncologia Sperimentale, Consiglio Nazionale Delle Ricerche, Napoli, Italy

**Keywords:** RASopathy, Autoimmunity, Immune system, CD8 T cells, Inflammatory cytokines

## Abstract

**Background:**

Abnormalities of the immune system are rarely reported in patients affected by RASopathies. Aim of the current study was to investigate the prevalence of immune system dysfunction in a cohort of patients affected by RASopathies.

**Study design:**

A group of 69 patients was enrolled: 60 at the Federico II University, Naples, 7 at University Magna Graecia of Catanzaro, 2 at “Scuola Medica Salernitana”, Salerno. An age- and sex-matched control group was also enrolled. Autoimmune disorders were investigated according to international consensus criteria. Immune framework was also evaluated by immunoglobulin levels, CD3, CD4, CD8, CD19, CD56 lymphocyte subpopulations, autoantibodies levels and panel of inflammatory molecules, in both patients and controls.

**Results:**

Frequent upper respiratory tract infections were recorded in 2 patients; pneumonia, psoriasis and alopecia in single patients. Low IgA levels were detected in 8/44 patients (18.18%), low CD8 T cells in 13/35 patients (37.14%). Anti-tg and anti-TPO antibodies were detected in 3/24 patients (12.5%), anti r-TSH in 2 cases (8.33%), all in euthyroidism. Serum IgA and CD8 levels were significantly lower in patients than in controls (p 0.00685; p 0.000656 respectively). All tested patients showed increased inflammatory molecules compared to controls. These findings may anticipate the detection of overt autoimmune disease.

**Conclusions:**

Patients affected by RASopathies are at risk to develop autoimmune disorders. Routine screening for autoimmunity is recommended in patients with RASopathy.

**Supplementary Information:**

The online version contains supplementary material available at 10.1186/s13023-021-02050-6.

## Introduction

RASopathies are a clinically defined group of medical genetic syndromes caused by germline mutations in the genes that encode components or regulators of the RAS/mitogen activated protein kinase (MAPK) pathway. Taken together, the RASopathies represent one of the most prevalent group of congenital malformation syndromes affecting approximately 1 in 1,000 individuals [1].

The RAS/MAPK pathway is a ubiquitous, highly conserved, intracellular signaling pathway that is critical in the cell cycle regulation, differentiation, growth, apoptosis and cell senescence [1]. Abnormalities in RAS expression, activation, and signaling pathways appear to play also an important role in the regulation of the inflammatory response and in autoimmune mechanisms [2–5].

RASopathies group include: Noonan syndrome (NS) caused by mutations in *PTPN11, SOS1*, *RAF1, KRAS, NRAS and CBL*; NS-like with loose anagen hair (NSLAH) due to germline mutations of *SHOC2* [6] or more rarely, *PPP1CB* [7]; NS with multiple lentigines (NSML) caused by specific mutations of *PTPN11* [8], although other rare mutations have been reported [9]; Costello syndrome (CS) caused by activating mutations in *HRAS*; cardio-facio-cutaneous syndrome (CFC) caused by gain of function mutations in *BRAF* and *MAP2K1* or *MAP2K2* [1]. Heterozygous missense mutations in *MAP2K1* (*MEK1*) and *MAP2K2* (*MEK2*) are present in approximately 25% of CFC individuals [10]. Mutations *RIT1* have been identified in 17 of 180 patients (9%) with Noonan syndrome or a related condition but with no detectable mutations in known Noonan-related genes [11]. *LZTR1* may be responsible of a rare percentage of NS cases [1].

RASopathies are multisystemic disorders with a unique phenotype, but they share many overlapping characteristics, including craniofacial dysmorphism, cardiac malformations, cutaneous, musculoskeletal, and ocular abnormalities, neurocognitive impairment; hypotonia and an increased cancer risk [12].

A RAS-associated autoimmune leukoproliferative disorder (RALD) has been described, characterized by a non-malignant clinical picture, partly overlapping to that of autoimmune lymphoproliferative syndrome (ALPS), represented by lymphadenopathy, splenomegaly, increased circulating B lymphocytes, hypergammaglobulinemia and autoimmunity. Unlike ALPS, RALDs do not generally show increased values of circulating double negative T lymphocytes, increased values of vitamin B12 or mutation of FAS, FASL or CASP10 [13].

Autoimmune diseases have rarely been described in NS. Case reports of patients with NS and autoimmune diseases such as systemic lupus erythematosus, celiac disease, Hashimoto thyroiditis [14] and chronic idiopathic thrombocytopenic purpura have been described [15]. Few cases of NS associated with autoimmune hepatitis have also been reported [16]. A cohort of patients with RASopathies including 42 patients showed a high frequency of positivity of autoantibody titers, in the presence or absence of associated clinical manifestations [17].

The aim of the present study was to perform immunological evaluation in a group of patients affected by RASopathies.

## Patients and methods

69 patients (43 males, 26 female) affected by RASopathy were enrolled in the study: 60 at the Pediatric Genetic Section of the University Federico II of Naples, 7 at the Department of Clinical and Experimental Medicine of the University Magna Graecia of Catanzaro and two at Department of Medicine, Surgery and Dentistry “Scuola Medica Salernitana”, Salerno (Italy). The protocol was discussed with each patient (or legal tutor) and informed consent was obtained.

Clinical diagnosis were: 61 NS, 5 CFC, 3 NSML. The mean age at the moment of the enrolment was 8.72 years, (ranges 0 to 26 years).

The enrollment was carried out according to the following inclusion criteria: (i) clinical diagnosis of RASopathy, based upon clinical features and confirmed by molecular analysis performed on DNA extracted from circulating leucocytes, (ii) informed consent expression. The exclusion criteria were: (i) denied consent to participate to the study.

The patients enrolled presented the following genetic mutations distribution: 56.52% *PTPN11*, 13.04% *SOS1*, 11.59% *BRAF*, 5.8% *RIT1*, 4.35% *LZTR1*, 2.9% *RAF1*, 1.45% *KRAS*, 1.45% *MAP2K2, 1.45*% *MEK1*.

50 age- and sex-matched healthy controls were also enrolled (30 males, 20 females) mean age 8.7 years (ranges 0–26).

This is a retrospective study: patients’ clinical data were obtained from medical records over the past 20 years. Moreover all patients (or legal tutor) underwent anamnestic recall, clinical examination, including auxological parameters. Clinical findings suggestive for infections disease or auto-immune disorder were recorded including: upper and lower airway infections, otitis, skin infection and/or presence of arthralgia, artritis, purpura. For all the categories, the type of defects and the frequency of the individual anomalies were analyzed. All autoimmune disorders were excluded or diagnosed in the study cohort according to international consensus criteria [18–24]. In all patients complete blood count, determination of C-reactive protein and thyroid profile were performed.

In a group of 44/69 patients and 30/50 controls quantitative analysis of immunoglobulin levels (IgA, IgG, IgM, IgE), was performed and interpreted according to the normal range (± 2DS) proposed by Ugazio et al. [25].

In a group of 35/69 patients and 50/50 controls CD3, CD4, CD8, CD19, CD56 lymphocyte subpopulations was performed by FACS and the normal range was considered according to the protocol provided by Dallavilla et al. [26].

Patients sample (24/69) were screened for antinuclear antibodies (ANA) by ELISA. Dilutions 1:320 were defined as positive. Anti Tg, anti-TPO, anti r-TSH anti-and LKM1 antibodies were assayed by ELISA. ENA and anti-dsDNA were measured by chemiluminescence. Rheumatoid factor (RF), anti-double-stranded DNA (anti-dsDNA), Anti-smooth muscle antibodies, anticardiolipin, Lupus anticoagulant, Anti-neutrophil cytoplasm antibody (ANCA), anti Tgasi, anti-beta 2 glycoprotein 1, glutamic acid anti-decarboxylase (GAD), anti-insulin (IAA), anti-tyrosine phosphatase (IA-2A) and anti-zinc transporter 8 were also detected. The serum levels of the C3 and C4 complement components were determined.

In a group of 10/69 patients and 10/50 controls available for further blood sampling, screening of a panel of inflammatory molecules was performed including PDGF, IL-1b, IL-1ra, IL-2, IL-4, IL-5, IL-6, IL-7, IL-8, IL-9, IL-10, IL-12(p70), IL-13, IL-15, IL-17, Eotaxin, FGF basic, G-CSF, GM-CSF, IFN-g, IP-10, MCP-1(MCAF), MIP-1a, MIP-1b, RANTES, TNF-a, VEGF. The tested molecules were chosen because they are important players in the pathogenesis of autoimmune disease.

### Statistical analysis

Each numerical variable is expressed as mean ± SD. Statistical analysis was performed using SPSS package.

Differences in the lymphocytes, autoantibodies and inflammatory molecules levels between patients and controls were analyzed using the *t-test* for unpaired data corrected for Fisher exact test. To investigate the presence of an association between severity of phenotype and either DNA mutation or specific gene involved, χ^2^ test was performed.

A P value < 0.05 was considered to be significant in all instances.

## Results

### Clinical parameters

All patients underwent anamnestic recall, clinical examination, including auxological parameters, clinical findings suggestive for infections or auto-immune disorder. Auxological parameters reveal short stature for specific growth chart in 21/69 patients.

Recurrent upper respiratory tract infections were recorded in 2 patients (2/69; 2.89%) and pneumonia in 1 patient (1/69, 1.45%).

In no case an autoimmune disorder was diagnosed, except for one case of psoriasis in a patient (1/69, 1.45%) with SOS1 mutation. Alopecia and leukemia were detected in a single patient (Table 1).

**Table 1:** Clinical and laboratory features of the patient cohort according to genetic mutation

Gene	PTPN11 (N = 40)	SOS1 (N = 9)	RAF1 (N = 2)	BRAF (N = 8)	LZTR1 (N = 3)	RIT1 (N = 4)	KRAS (N = 1)	MAP2K2 (N = 1)	MEK1 (N = 1)
Infection in regions including the middle ear and upper airway tract	1	1	–	–	–	–	–	–	–
Pneumonia	1	–	–	–	–	–	–	–	–
Decreased IgA	4	1	–	2	–	1	–	–	–
Decreased IgG	1	1	–	1	–	–	–	–	–
Decreased IgM	1	1	–	1	–	1	–	–	–
< CD3	1	–	–	–	–	–	–	–	–
< CD8	2	2	–	1	–	1	–	–	–
Anti Tg	1	–	–	1	1	1	–	–	–
Anti–TPO	1	–	–	1	1	–	–	–	–
Anti R–TSH	2	–	–	–	–	–	–	–	–
Anti–LKM1	1	–	–	–	–	–	–	–	–
ENA/Anti–dsDNA	–	–	–	–	–	–	–	–	–
Autoimmune diseases	–	1 Psoriasis	–	–	–	–	–	–	–

### Biochemical parameters

Immunoglobulin data, lymphocyte classes and inflammatory molecules were analysed in patients and controls. IgA levels were lower in patients than in controls (p 0.00685) (Table 2(a)). Patients showed lower CD8 than controls (p 0.000656) (Table 2(b)).

**Table 2:** (a) Mean of immunoglobulins values in patients and controls. (b) Main lymphocyte subpopulations

	Patients (N = 44)	Controls (N = 30)	P
	Mean ± DS	Mean ± DS	
(a)			
IgA	96.35294 ± 49.40044	140.25 ± 34.19762	0.00685
IgG	948 ± 217.1421	866.2667 ± 324.5688	0.219003
IgM	103.027 ± 43.5993	135.4667 ± 32.5727	0.012328

	Patients (N = 35)	Controls (N = 50)	P
	Mean ± DS	Mean ± DS	
(b)			
CD3	1887 ± 1279.5	1924.74 ± 558.7	0.854889
CD4	1276.18 ± 1072.57	1026.16 ± 295.53	0.120953918
CD8	502.84 ± 281.1	707.4 ± 232.7	0.000656
CD19	536.76 ± 412.71	403.82 ± 301.49	0.093913361
CD56	328.57 ± 311.12	323.98 ± 129.34	0.92731834

Inflammatory molecules were significantly higher in patients than in controls: IL1-ra (0.002646), IL-2 (p 0.027678), IL-4 (p 0.017983), IL-6 (p 0.033026). IL-7 (p 0.012856). IL-10 (p 0.014939). IL-15 (p 0.01665). Eotaxin (p 0.000724). G-CSF (p 0.014625). IP-10 (0.029932) (Table 3).

**Table 3:** Mean of cytokine values in patients and controls

	Patients (N = 10)	Controls (N = 10)	P
	Mean ± DS	Mean ± DS	
IL1-ra	840.1 ± 278.9	426.4 ± 251.3	0.002646
IL-2	18.8 ± 6.63	13.72 ± 1.32	0.027678
IL4	6.1 ± 2.90	3.715 ± 0.54	0.017983
IL6	13.41 ± 7.19	7.998 ± 1.799	0.033026
IL-7	46.62 ± 9.37	37.96 ± 3.24	0.012856
IL-10	14.97 ± 4.095	10.89 ± 2.49	0.014939
IL-15	439.42 ± 112.26	324.05 ± 80.61	0.01665
Eotaxin	121.43 ± 32.94	71.757 ± 20.16	0.000724
G-CSF	371.69 ± 221.266	177.45 ± 52.57	0.014625
IP-10	798.15 ± 247.03	575.96 ± 166.79	0.029932

Altered values of Ig levels were recorded in 10/44 patients, 22.72%. Low IgA levels were found in 8/44 patients, 18.18% (Additional file 1: Table S1). Two of them showed recurrent upper respiratory tract infections.

**Fig. 1:**
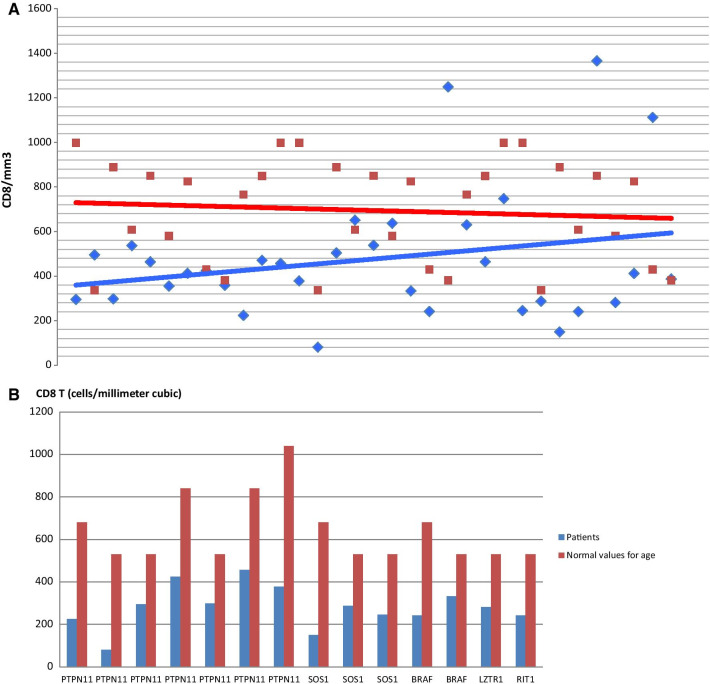
**A** CD8 T cells values in patients (blue diamond) and controls (red square). **B** CD8 T cells values in patients compared to normal value for age

Decreased values of CD3 and/or CD8 were recorded in 14/35 patients (40%). CD19 was reduced in 4 cases (4/35, 11.43%) (Additional file 1: Table S2), Fig. 1.

One of the patients with PTPN11 mutation and a reduction in the TCD8 value also had a reduction in IgA and IgG.

A total of 6 patients out of 24 (25%) presented with at least one autoantibody described below. Four presented with the concomitant occurrence of two autoantibodies. Anti-tg were detected in 4/24 patients (16.6%), 3 of these (12.5%) also showed anti-TPO antibodies. Anti r-TSH in 2/24 patients (8.33%), all in euthyroidism. The presence of Anti-LKM1 was detected in one patient (4.16%) who showed anti r-TSH also.

Screening of a panel of inflammatory molecules revealed a significant increase of IL(Interleukin)-1ra, IL-2, IL-4, IL-6, IL-7, IL-10, IL-15, Eotaxin, G-CSF and IP-10, in all patients tested (Table 3).

No association between gene involved and biochemical parameters was recorded.

## Discussion

Abnormalities of the immune system or autoimmune diseases are rarely reported in patients affected by RASopathies. The current retrospective study performed immunological investigation in a cohort of 69 patients and 50 controls.

The results of the current study showed lower IgA levels in patients than in controls with a prevalence of 18% of IgA deficiency in patients group. The worldwide prevalence of selective IgA deficiency depends on the ethnic background and it is most prevalent in Caucasians (1:600) [27]. Most individuals are asymptomatic, but the defect may be associated with recurrent respiratory and gastrointestinal tract infections/disorders, autoimmunity and allergies [28, 29]. Recurrent upper respiratory tract infections were recorded in patients with IgA deficiency in the current study. We suggest to investigate immunoglobulin serum levels in patients affected by RASopathies.

Recently, a cohort of 42 patients with RASopathies was evaluated for autoimmune status. Autoimmune antibodies were observed in 52% of the patients. Remarkably, three (7%) of the patients had specific gastrointestinal and liver autoantibodies without clinical findings. Six patients (14%) fulfilled the clinical criteria for autoimmune diseases [systemic lupus erythematous, polyendocrinopathy (autoimmune thyroiditis and celiac disease), primary antiphospholipid syndrome, autoimmune hepatitis, vitiligo, and autoimmune thyroiditis] [17]. Other cases of autoimmune diseases are reported anecdotally in patients with Rasopathies [2, 30–33].

Although clinical findings suggestive for autoimmune disease were detected in only one patient of the current case load, biochemical parameters showed specific alterations.

Our study has highlighted the frequent finding of thyroid autoantibodies (25%), all in condition of euthyroidism, as already reported [34]. In recent years, numerous prospective studies have demonstrated that many autoantibodies can be detected in the serum of asymptomatic or paucisymptomatic individuals who later develop an autoimmune disease. These antibodies can therefore precede the clinical symptoms of the disease by years, and could in principle be used for diagnostic and prognostic purposes, including screening studies [35].

Reduced CD8+ T-cells levels were also demonstrated in our patients. Although the role of CD8+ T cells is not as well established, it is known that CD8+ T cells contribute to the induction, progression, pathogenesis and protection from many autoimmune diseases [36–38].

As known, Ras/MAPK signalling is also implicated in peripheral tolerance to prevent autoimmune destruction by self-reactive T cells that escape thymic deletion. In particular, Erk MAPK pathway plays a critical role in CD8 T cell activation, proliferation, and survival [39].

On the basis of data reported in literature, it might be suggested that impairment of RAS-MAPK pathway alters CD8 production causing intolerance and cross reactivity. Other studies are needed to confirm these hypotheses.

We hypothesized that reduced CD8+ T-cells levels might be the first detectable sign of possible emergence of autoimmune disease.

On the other hand, cytokines including proinflammatory cytokines (IL-1, TNFα, IFN, IL-2, IL-6, IL-12) and consequently anti-inflammatory cytokines (IL-10, IL-11, IL-13, IL-1ra) are important players in the pathogenesis of autoimmune disease through multiple ways, such as regulating inflammation and angiogenesis [40, 41].

It is interesting that in all the studied patients high levels of cytokines were recorded. Patients described in the current study showed high levels of IL-4, known to be involved in the development of autoantibodies and autoantibody mediated diseases [42]. Even more important, IL-6 is a critical cytokine that mediates numerous inflammatory and immunomodulatory pathways. In this regard, dysregulated and persistent IL-6 production results in severe inflammatory and autoimmune disorders [43]. An increase in cytokine with the key role in anti-inflammatory response, IL10, or of maintaining self-tolerance, IL2, was also demonstrated [44, 45]. It might be suggested that the increase of inflammatory molecules levels with a state of chronic low-grade inflammation represents the underlying pathological mechanism leading to autoimmune diseases in this group of patients.

In conclusion, the results of the current study suggested a tendency to autoimmune phenomena as demonstrated by the finding of circulating autoantibodies, low levels of CD8 T cells and high levels of inflammatory cytokines. These evidences may be the first markers of the possible evolution to overt autoimmune disease.

## Limits of study

The main limit of the study is that not all the patients were available for all the tests and therefore the conclusions are partial.

Moreover, the average age of our patients is relatively low, which probably limits the diagnosis of autoimmune disorders that have a later onset.

## Conclusion

Limited to tested patients with RASopaties, this study shows high prevalence of IgA deficiency, low TCD8 lymphocytes count and high inflammatory molecules levels. The detection of autoantibodies may anticipate the detection of overt autoimmune disease.

A comprehensive clinical and biochemical assessment should be carried out both at diagnosis and during the follow-up. We suggest the importance to include the dosage of serum immunoglobulins, and lymphocyte classes among the annual screening tests performed in this group of patients. The cytokine assay, on the other hand, could be more useful for research purposes. A correct endocrinological follow-up with thyroid profile is worthwhile, considering the high prevalence of positivity for autoantibodies.

In order to recommend routine screening for autoimmunity in patients with asymptomatic RASopathy, continuous monitoring will be required for possible emergence of autoimmune disease. Other studies are also needed to confirm our data.

## Supplementary Information

**Additional file 1: Table S1**. Patients immunoglobulin values compared to normal values for age. **Table S2**. Patients lymphocyte subpopulations values compared to normal values for age. Normal values are reported as mean (10th and 90th percentile). *Taken from Shearer et al, JACI 2003*.

## Data Availability

Data are available by request.

## Abbreviations

MAPK: Mitogen activated protein kinase
NS: Noonan syndrome
NSLAH: NS-like with loose anagen hair
CS: Costello syndrome
CFC: Cardio-facio-cutaneous syndrome
RALD: RAS-associated autoimmune leukoproliferative disorder
ALPS: Autoimmune lymphoproliferative syndrome
NF1: Neurofibromatosis type 1
