# Supplementary material for: Risk of autoimmune diseases in patients with RASopathies: systematic study of humoral and cellular immunity
Source: Orphanet J Rare Dis. 2021 Oct 2;16:410. doi: 10.1186/s13023-021-02050-6 (PMC8487584; doi:10.1186/s13023-021-02050-6)
Supplement: Supplementary file 1 — Additional file 1: Table S1. Patients immunoglobulin values compared to normal values for age. Table S2. Patients lymphocyte subpopulations values compared to normal values for age. Normal values are reported as mean (10th and 90th percentile). Taken from Shearer et al, JACI 2003. [file 13023_2021_2050_MOESM1_ESM.docx]

| **Supplementary Table S1. Patients immunoglobulin values compared to normal values for age** | | | | | | | | |
| --- | --- | --- | --- | --- | --- | --- | --- | --- |
| **Gene**  **(10/44)** | **Age** | **Infections disease** | **IgA**  **(mg/dl)** | **IgG**  **(mg/dl)** | **IgM**  **(mg/dl)** | **Normal values. Mean (range) (mg/dl)^25^** | | |
|  | | | | | | **IgA** | **IgG** | **IgM** |
| **PTPN11** | 8 y | No | 104 | 1200 | **48.3** | 113 (31-315) | 1164 (462-1682) | 121 (56-261) |
| **PTPN11** | 17 y | Yes | **35.5** | 1110 | 86.1 | 136 (61-301) | 1105 (604-1909) | 132 (59-297) |
| **PTPN11** | 4 y | No | **28.5** | **495** | 68.2 | 98 (37-257) | 1117 (528-1959) | 119 (49-292) |
| **PTPN11** | 16 y | No | **34.1** | 976 | **58.4** | 136 (61-301) | 1105 (604-1909) | 132 (59-297) |
| **PTPN11** | 7 y | No | **28.5** | 831 | 92.6 | 113 (31-315) | 1164 (462-1682) | 121 (56-261) |
| **SOS1** | 9 y | Yes | **36.9** | **626** | 72.7 | 127 (60-270) | 1164 (707-1919) | 129 (61-276) |
| **SOS1** | 4 y | No | 100 | 937 | **41.1** | 98 (37-257) | 1117 (528-1959) | 119 (49-292) |
| **BRAF** | 2 y 3 m | No | **<27.9** | 506 | **48.9** | 68 (27-173) | 889 (462-1710) | 126 (62-257) |
| **BRAF** | 10 y 6m | No | **53** | **614** | 64 | 127 (60-270) | 1164 (707-1919) | 129 (61-276) |
| **RIT1** | 16 y | No | **<6.5** | 968 | **22** | 136 (61-301) | 1105 (604-1909) | 132 (59-297) |

| **Supplementary Table S2. Patients lymphocyte subpopulations values compared to normal values for age. Normal values are reported as mean (10th and 90th percentile)^46^** | | | | | | | | | | | | |
| --- | --- | --- | --- | --- | --- | --- | --- | --- | --- | --- | --- | --- |
| **Gene N=16/35** | **Age** | **Ly.**  **(/µL)**  **(%)** | **CD3**  **(/µL)**  **(%)** | **CD4**  **(/µL)**  **(%)** | **CD8(/µL)**  **(%)** | **CD19**  **(/µL)**  **(%)** | **CD56**  **(/µL)**  **(%)** | **Normal values** | | | | |
|  | | | | | | | | **CD3** | **CD4** | **CD8** | **CD19** | **CD56** |
| **PTPN11** | 7 y | 1870 | 1159  62% | **673**  36% | **224**  12% | 411  22% | 150  8% | 1820 (1200-3700) | 980 (650-1500) | 680 (370-1100) | 480 (270-860) | 230 (100-480) |
| **PTPN11** | 13 y | 1150 | **518**  45% | **402**  35% | **81**  7% | **265**  23% | 265  23% | 3550 (2100-6200) | 2160 (1300-3400) | 530 (330-920) | 1310 (720-2600) | 360 (180-920) |
| **PTPN11** | 17 y | 1640 | 1099  67% | 672  41% | **295**  18% | 328  20% | 164  10% | 1480 (1000-2200) | 840 (530-1300) | 530 (330-920) | 300 (110-570) | 190 (70-480) |
| **PTPN11** | 4 y | 1570 | **958**  61% | **534**  34% | **424**  27% | **188**  12% | 361  23% | 2930 (1400-3700) | 1380 (700-2200) | 840 (490-1300) | 750 (390-1400) | 300 (130-720) |
| **PTPN11** | 17 y | 1420 | **880**  62% | **469**  33% | **298**  21% | 256  18% | 128  10% | 1480 (1000-2200) | 840 (530-1300) | 530 (330-920) | 300 (110-570) | 190 (70-480) |
| **PTPN11** | 3 y | 3260 | 2152  66% | 1467  45% | **457**  14% | **489**  15% | 326  10% | 3550 (2100-6200) | 2160 (1300-3400) | 840 (490-1300) | 1310 (720-2600) | 360 (180-920) |
| **PTPN11** | 2 y | 3820 | 2131  56 % | **989**  26% | **378**  20% | **669**  18% | 737  19% | 3550 (2100-6200) | 2160 (1300-3400) | 1040 (620-2000) | 1310 (720-2600) | 360 (180-920) |
| **SOS1** | 10 y | 1350 | **783**  58% | **405**  30% | **149**  11% | 338  25% | 176  13% | 1820 (1200-3700) | 980 (650-1500) | 680 (370-1100) | 480 (270-860) | 230 (100-480) |
| **SOS1** | 15 y | 2610 | 1435  55% | 992  38% | **287**  11% | 600  23% | 209  8% | 1480 (1000-2200) | 840 (530-1300) | 530 (330-920) | 300 (110-570) | 190 (70-480) |
| **SOS1** | 26 y | 1360 | **940**  n.a. | 626  n.a. | **245**  n.a. | 136  n.a. | 218 | 1480 (1000-2200) | 840 (530-1300) | 530 (330-920) | 300 (110-570) | 190 (70-480) |
| **BRAF** | 6 m | 10410 | 6805  66% | 4997  48% | 1249  12% | 1666  16% | **1667**  16% | 3930 (2500-5600) | 2850 (1800-4000) | 1050 (590-1600) | 1550 (430-3000) | 420 (170-830) |
| **BRAF** | 9 y | 1420 | **839**  59% | **426**  30% | **241** 17% | 213 15% | 227  16% | 1820 (1200-3700) | 980 (650-1500) | 680 (370-1100) | 480 (270-860) | 230 (100-480) |
| **BRAF** | 15 y | 2460 | 1451  59% | 836  34% | 333  13% | 197  8% | **812**  33% | 1480 (1000-2200) | 840 (530-1300) | 530 (330-920) | 300 (110-570) | 190 (70-480) |
| **LZTR1** | 16 y | 1860 | 1302  70% | 633  34% | **281**  15% | 299  16% | 168  9% | 1480 (1000-2200) | 840 (530-1300) | 530 (330-920) | 300 (110-570) | 190 (70-480) |
| **RIT1** | 16 y | 1420 | **895**  63% | 596  42% | **241**  17% | 270  19% | 114  8% | 1480 (1000-2200) | 840 (530-1300) | 530 (330-920) | 300 (110-570) | 190 (70-480) |
| Ly: lymphocytes; n.a.: not avaible; | | | | | | | | | | | | |
